# Supplementary material for: Development of a computer-aided design software for the quantitative evaluation of aesthetic damage
Source: PLoS One. 2019 Dec 18;14(12):e0226322. doi: 10.1371/journal.pone.0226322 (PMC6919621; doi:10.1371/journal.pone.0226322)
Supplement: S1 Code site — 1. (ZIP) [file pone.0226322.s001.zip › [EESC_jr][Projeto]Relatorio_07_21/reports/templates_zip/gentelella-master/vendors/morris.js/examples/timestamps.html]

# Timestamps

```
/* data stolen from http://howmanyleft.co.uk/vehicle/jaguar_'e'_type */
var timestamp_data = [
  {"period": 1349046000000, "licensed": 3407, "sorned": 660},
  {"period": 1313103600000, "licensed": 3351, "sorned": 629},
  {"period": 1299110400000, "licensed": 3269, "sorned": 618},
  {"period": 1281222000000, "licensed": 3246, "sorned": 661},
  {"period": 1273446000000, "licensed": 3257, "sorned": 667},
  {"period": 1268524800000, "licensed": 3248, "sorned": 627},
  {"period": 1263081600000, "licensed": 3171, "sorned": 660},
  {"period": 1260403200000, "licensed": 3171, "sorned": 676},
  {"period": 1254870000000, "licensed": 3201, "sorned": 656},
  {"period": 1253833200000, "licensed": 3215, "sorned": 622}
];
Morris.Line({
  element: 'graph',
  data: timestamp_data,
  xkey: 'period',
  ykeys: ['licensed', 'sorned'],
  labels: ['Licensed', 'SORN'],
  dateFormat: function (x) { return new Date(x).toDateString(); }
});
```
